# Event-based surveillance in Republic of Korea: assessment of the effectiveness of Epidemic Intelligence from Open Sources

**DOI:** 10.5365/wpsar.2025.16.1151

**Published:** 2025-07-28

**Authors:** Seontae Kim, Jia Lee, Jiyoung Oh, Ji Joo Lee, Geehyuk Kim, Jaehwa Chung, Yunhee Lee, Yongmoon Kim, Sangwoo Tak

**Affiliations:** a Division of Risk Assessment, Bureau of Public Health Emergency Preparedness, Korea Disease Control and Prevention Agency, Cheongju, Republic of Korea.

## Abstract

In 2023, Republic of Korea’s Korea Disease Control and Prevention Agency (KDCA) enhanced its event-based surveillance practices by using the World Health Organization’s (WHO) Epidemic Intelligence from Open Sources (EIOS) to actively screen and share information about potential public health threats to the country. This report describes the preliminary assessment of the results of implementing these enhanced event-based surveillance activities from June to October 2023. During this period, 425 (0.4%) events were detected globally by the KDCA from 99 945 media articles, with the highest frequency reported in Asia (185, 43.5%) and North America (81, 19.1%). The most frequently reported diseases or conditions were dengue fever (111, 26.1%) and mpox (32, 7.5%). Eight events were detected early by the KDCA using EIOS before being officially listed on WHO’s Event Information Site (EIS) or in Disease Outbreak News (DON), with an average interval of 20 days (range: 5–41) between the detection date and posting on EIS or DON. Thus, EIOS is efficient in aiding early detection of potential public health threats at the national level. This finding highlights the importance of sustaining international cooperation and support to enhance surveillance capabilities in resource-limited settings and expanding the scope of EIOS, including by incorporating additional sources and sources in additional languages, reducing noise. However, as the current report is based on a descriptive analysis, in the future a systematic evaluation of event-based surveillance using EIOS to identify relevant attributes will need to be conducted.

Event-based surveillance (EBS) is the organized and rapid capture of information about events that are a potential risk to public health. (*1*) This information includes rumours and other ad hoc reports transmitted through formal channels (e.g. established routine reporting systems) and informal channels (e.g. media). Information obtained through EBS should be rapidly assessed for its potential impact on public health risks, and appropriate responses should be undertaken. The World Health Organization (WHO) has emphasized the importance of developing and implementing EBS systems, alongside indicator-based systems, to rapidly detect public health emergencies and gather information for risk assessments, while adhering to commitments outlined in the International Health Regulations (2005; IHR). (*2*)

In 2017, WHO launched the Epidemic Intelligence from Open Sources (EIOS) initiative in collaboration with global public health stakeholders. (*3*) This system collates and categorizes articles daily from various open sources using text mining and analytical modules. The open sources include traditional online media, government and other official web sites, and existing web-based EBS tools, such as the Global Public Health Intelligence Network (GPHIN). EIOS has been widely used nationally and internationally to enhance capacities for the early detection of events and to facilitate EBS activities.

In Republic of Korea, the Korea Disease Control and Prevention Agency (KDCA) joined the EIOS community after the EIOS Global Technical Meeting held in Seoul in 2019. Since then, EIOS has been used on an ad hoc basis to support EBS activities, including active screening and for sharing information about events that have the potential to pose public health threats to the country, such as threats from emerging infectious diseases and unknown pathogens, and events arising from international mass gatherings (*4*) and new variants of COVID-19. Following standard operating procedures for EBS and risk assessments developed by the Division of Risk Assessment, EBS activities at the KDCA were enhanced in 2023 through the routine utilization of EIOS. On 16 May 2023, the Division of Risk Assessment established a daily routine surveillance dashboard within EIOS, using selected categories and sources, to assess the effectiveness of EIOS in detecting public health threats, with a focus on infectious diseases. This report provides an overview of the use of the dashboard and describes a preliminary assessment of using EIOS for EBS activities at the KDCA from 1 June to 31 October 2023. The report highlights the effectiveness of EIOS in aiding in the early detection of potential public health threats caused by infectious diseases at the national level.

## Methods

### Data collection process

**Supplementary Fig. 1** illustrates the process of EBS activities at the KDCA while monitoring EIOS, including detecting, filtering and verifying information about potential events, as well as conducting daily assessments and weekly discussions, and disseminating information about them. First, detection is conducted daily at the Division by eight trained personnel to capture unstructured information from combined sources, including both the EIOS dashboard (i.e. the daily routine surveillance dashboard) and conventional sources, which include media and other web-based sites that monitor infectious diseases, national health-related ministries or authorities, and official letters from embassies. Second, filtering is performed to screen the information according to selection criteria, such as determining whether there is an unusual or unexpected event or clustered morbidity and mortality (**Supplementary Box 1**). If information might be relevant to more than one of the selection criteria, it is defined as a signal. Third, verification and daily assessments are carried out by cross-checking the validity of the signal with multiple sources, including by collecting reliable additional information, from sources such as press releases or official statements, as well as direct communication through official letters from embassies and IHR National Focal Points. Once verified, a signal is classified as an event – that is, it may be a public health threat to Republic of Korea. Fourth, weekly discussions are conducted by the Division to determine which events should be communicated based on their urgency and priority, and detailed information is then disseminated within or outside the KDCA accordingly.

### Data sources

We used the EBS database, managed by the Division of Risk Assessment. Once a signal is identified as an event through the verification and daily assessment processes, information about an event is recorded, such as the date of detection, event name, affected continents and countries, information sources, event description and modes of communication. To compare the EBS database at the KDCA with information from WHO, data were extracted about events officially posted between 1 June and 31 October 2023 on the WHO Event Information Site (EIS) for IHR National Focal Points and in Disease Outbreak News (DON) as of 5 January 2024, including the posting date, event name and affected countries.

### Data analysis

The number of signals (i.e. information screened through initial filtering by trained personnel that might be relevant to the selection criteria) and events identified (i.e. signals that have the potential to pose public health threats, as evaluated through verification and daily assessments) and communication through EBS activities using EIOS were described for the period between 1 June and 31 October 2023.

A descriptive analysis of events recorded in the EBS database at the KDCA was carried out. The frequency of events by continent and the 10 most common diseases or conditions by month were analysed. Ongoing events were included if they were related to events previously documented in the EBS database. Additionally, a manual review of data about events on EIS and DON was undertaken to identify events that corresponded to those detected by the KDCA through its EBS activities using EIOS, and the estimated intervals were calculated between the detection date via the KDCA’s EBS and the posting date on EIS or on DON. All analyses were conducted using a Microsoft Excel 2021 spreadsheet.

## Results

### Detection and communication of events

From 1 June to 31 October 2023, a total of 99 945 pieces of information were collected on the EIOS dashboard, excluding data scanned from conventional sources, of which 2844 (2.8%) signals met the selection criteria. Following verification and daily assessment of all identified signals, 425 (0.4%) events detected globally had the potential to pose a public health threat to Republic of Korea. The number of detected events varied slightly by month, ranging from 72 (16.9%) in September and October to 106 (24.9%) in June (**Table 1**). Among the 425 events detected, information about 35 (8.2%) was shared internally with relevant divisions within the KDCA through brief situation analyses or weekly restricted reports about global infectious diseases; information about 96 (22.6%) events was disseminated externally, based on urgency and priority, with information about 51 (53.1%) of these disseminated through weekly open-access reports about global infectious diseases, information about 37 (38.5%) disseminated through online travel health advice platforms and information about 8 (8.3%) disseminated through monthly newsletters for health-care workers (**Table 1**).

**Table 1:** Number of signals and events identified, and instances of communication conducted through event-based surveillance activities using Epidemic Intelligence from Open Sources, by month, Korea Disease Control and Prevention Agency, June–October 2023

Variable	Information^a^	Signals^b^	Events^c^	Communication^d^	
				Internal^e^	External^f^
**Total**	**99 945 (100.0)**	**2844 (100.0)**	**425 (100.0)**	**35 (100.0)**	**96 (100.0)**
**June**	**19 001 (19.0)**	**717 (25.2)**	**106 (24.9)**	**6 (17.1)**	**19 (19.8)**
**July**	**18 441 (18.5)**	**542 (19.1)**	**87 (20.5)**	**5 (14.3)**	**16 (16.7)**
**August**	**20 934 (20.9)**	**590 (20.7)**	**88 (20.7)**	**9 (25.7)**	**14 (14.6)**
**September**	**20 675 (20.7)**	**546 (19.2)**	**72 (16.9)**	**6 (17.1)**	**24 (25.0)**
**October**	**20 894 (20.9)**	**449 (15.8)**	**72 (16.9)**	**9 (25.7)**	**23 (24.0)**
**Average no. per working day ** **(*n* = 153 days)**	**653.2**	**18.6**	**2.8**	**0.2**	**0.6**

Data are presented as *n*(%).

^a^ Information comprises articles pulled into the EIOS dashboard, excluding data scanned from conventional sources.

^b^ Signals are defined as information screened by initial filtering by trained personnel that might be relevant to the selection criteria.

^c^ Events are defined as signals identified through verification and daily assessment that have the potential to pose public health threats to Republic of Korea.

^d^ Communication is defined as the sharing of detailed information about events that have been determined through weekly discussions to require dissemination, based on urgency and priority.

^e^ Internal communication includes sharing brief situation analyses and weekly restricted reports about global infectious diseases with relevant divisions within the Korea Disease Control and Prevention Agency.

^f^ External communication includes weekly sharing of open-access reports about global infectious diseases (e.g. https://dportal.kdca.go.kr/pot/bbs/BD_selectBbsList.do?q_bbsSn=1009, in Korean) and monthly newsletters for health-care workers (e.g. http://kdcanewsletter.or.kr, in Korean), as well as updating online travel health advice platforms (e.g. http://xn--now-po7lf48dlsm0ya109f.kr/nqs/oidnow/main.do, in Korean).

### Events by continent and disease or condition

**Fig. 1:**
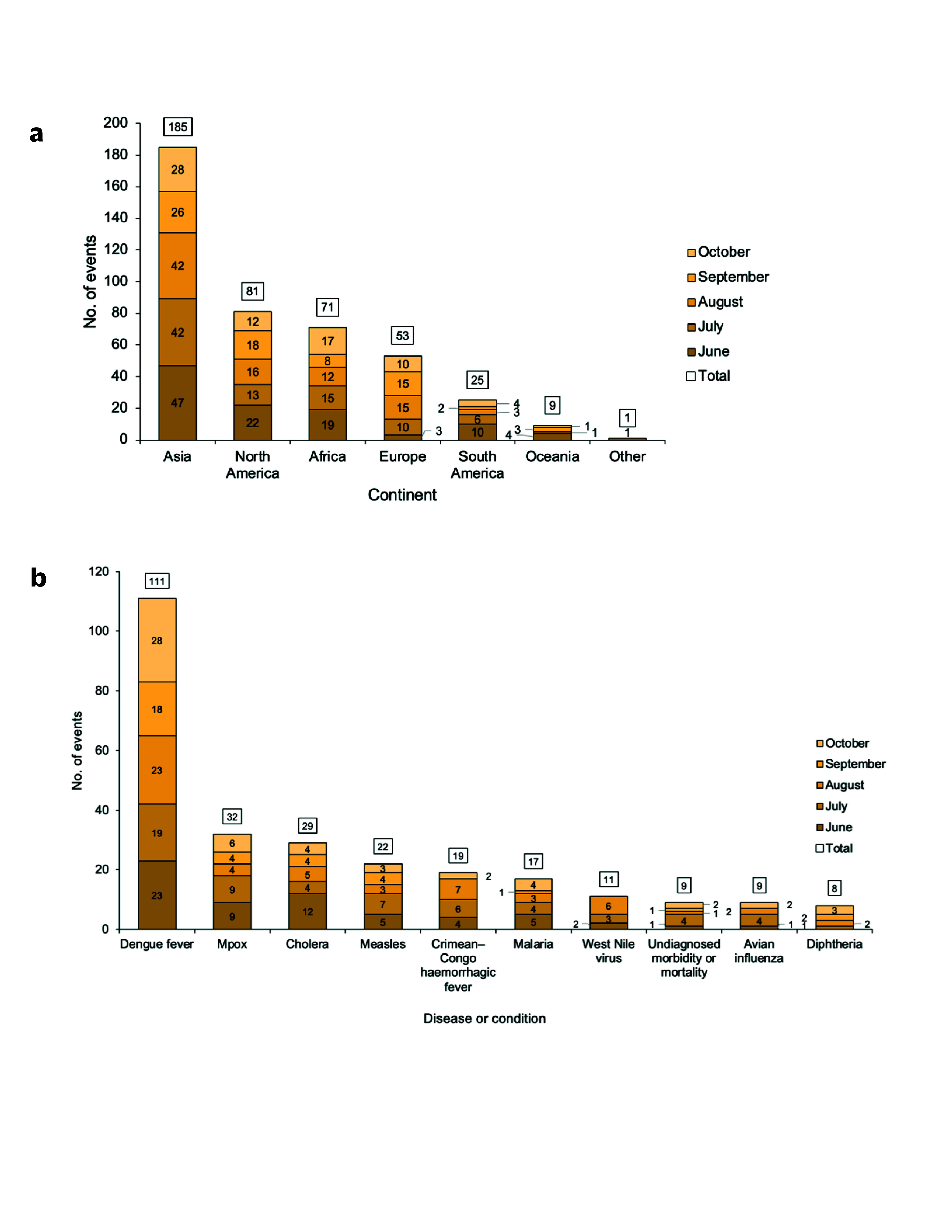
(a) Number of events by continent and (b) 10 most frequently reported diseases or conditions identified through event-based surveillance activities using Epidemic Intelligence from Open Sources, by month, Korea Disease Control and Prevention Agency, June–October 2023a

The total number of events during the study period varied by continent. The most frequently affected continent was Asia, reporting 185 (43.5%) events. The North American continent reported 81 (19.1%), followed by Africa with 71 (16.7%), Europe with 53 (12.5%) and South America with 25 (5.9%). The fewest events were reported in Oceania (9, 2.1%). Among the 425 global events, the most frequently reported disease was dengue fever, accounting for 111 (26.1%) events, followed by mpox (32, 7.5%) and cholera (29, 6.8%). Other frequently reported diseases or conditions included measles (22, 5.2%), Crimean–Congo haemorrhagic fever (19, 4.5%) and malaria (17, 4.0%) (**Fig. 1**). Additionally, the majority of events were reported by GPHIN and through the EIOS dashboard (313, 73.6%) (**Supplementary Table 1**).

[**insert** Figure 1]

### Review of events

After reviewing the data extracted from EIS and DON, we identified 15 events corresponding to those recorded in the EBS database at the KDCA among the events posted on EIS from 1 June to 31 October 2023; five events were excluded as they were not recognized as events by the KDCA, despite being posted by WHO. Of the 15 identified events, seven documented in the EBS database at the KDCA were originally detected through EIS; these included six influenza events identified as being an avian or animal influenza virus from Brazil, China, Kingdom of the Netherlands, United Kingdom of Great Britain and Northern Ireland, and United States of America, as well as one event associated with vaccine-derived poliovirus type 2 from United Republic of Tanzania. The remaining eight events were detected early through EBS using the EIOS dashboard at the KDCA before being officially listed on EIS or DON. The average interval between the detection date via EBS at the KDCA and the posting date on EIS or DON for the eight events was approximately 20 days (range: 5–41) (**Table 2**).

**Table 2:** Characteristics of eight events detected early through event-based surveillance activities using Epidemic Intelligence from Open Sources at the Korea Disease Control and Prevention Agency, compared with the time events were posted on the World Health Organization’s Event Information Site and Disease Outbreak News site, June–October 2023

Detection date at KDCA^a^	Event^b^	Country	EIS posting date	DON posting date	Interval (days)^c^
**29 June**	**Guillain–Barré syndrome**	**Peru**	**16 July**	**25 July**	**17**
**18 July**	**Dengue fever**	**Bangladesh**	**4 August**	**11 August**	**17**
**20 July**	**Dengue fever**	**Egypt**	**9 August**	**NA**	**20**
**21 July**	**Avian influenza**	**China**	**26 July**	**NA**	**5**
**31 August**	**Dengue fever**	**Chad**	**11 October**	**16 October**	**41**
**4 September**	**Diphtheria**	**Guinea**	**12 October**	**18 October**	**38**
**12 September**	**Nipah virus infection**	**India**	**28 September**	**3 October**	**16**
**13 September**	**Botulism**	**France**	**NA**	**20 September**	**7**

DON: Disease Outbreak News; EIS: Event Information Site; KDCA: Korea Disease Control and Prevention Agency; NA: not applicable.

^a^ The detection date is the date when signals were detected through event-based surveillance activities using the EIOS dashboard by trained personnel at the KDCA.

^b^ The following events are not included in this table: seven events of avian or animal influenza virus originally detected through EIS, as well as five events posted on EIS that were not identified as events by the KDCA from June to October 2023.

^c^ The interval represents the number of days between the detection date at the KDCA and the posting date on either EIS or DON, whichever was earlier.

## Discussion

Through EBS activities conducted using an EIOS dashboard at the KDCA, a total of 425 events were detected between 1 June and 31 October 2023. Dengue fever accounted for the highest proportion of diseases, followed by mpox. The global upsurge in dengue cases in 2023 compared with prior years was consistently reported, and is partly due to climate change. (*5*, *6*) In 2023, several local transmission events of dengue were reported in China, Taiwan (China) in June; (*7*) Chad in August; (*8*) and in France, Italy and Spain from June to November. (*6*) Although dengue is not endemic in Republic of Korea, and most cases involve travellers infected outside the country, the occurrence of viraemic travel-related cases underscores the potential for local transmission, especially considering the rapid increase in dengue cases globally and in international travel. Therefore, pre-emptive surveillance measures for imported cases remain pivotal to prevent local transmission of dengue virus in the future, (*9*) as the vector (*Aedes albopictus*) is widespread across the country. In Asia and North America, the continents with the most reported events, the United States, China, Taiwan (China) and India had the highest counts (data not shown).

Although the types of diseases and conditions reported varied, the majority of those reported from China, Taiwan (China) were dengue fever, likely influenced by regular updates on the dengue fever outbreak provided by the government following the first local case in June 2023. (*7*) Hence, caution needs to be exercised when interpreting the results of ongoing events in EBS databases.

This report has at least two limitations. First, detected events were categorized based on unstructured information, including outbreak reports or media articles from the EIOS dashboard and conventional sources; thus, the frequency of events does not indicate the number of confirmed cases. Second, data were not systematically analysed about ongoing events related to events previously documented in the EBS database; therefore, the number of ongoing events might have been overestimated.

The low number or absence of events in parts of Africa and East Asia may reflect limited surveillance and laboratory capacity for disease detection and notification in low- and middle-income countries or challenges regarding language barriers within EIOS. Ganser et al. identified global disparities in EBS performance for disease outbreak detection, with high-income countries demonstrating optimal performance. (*10*) Additionally, only five Member States from the WHO Western Pacific Region – China, Japan, the Philippines, Republic of Korea and Singapore – had joined the EIOS community as of 12 December 2023. (*11*) These observations emphasize the significance of sustaining international cooperation and support to enhance surveillance capabilities in resource-limited settings and expanding the scope of EIOS, including by incorporating additional sources and languages. This will contribute to improving the completeness of EBS when using EIOS, thus enabling early detection and prompt response to public health threats worldwide.

The average interval between the time an event was detected through the KDCA’s EBS activities using EIOS and the posting date for the event on EIS or DON for eight events was approximately 20 days. Original sources of information pooled into the EIOS dashboard for those events included three media articles from GPHIN. These results support the utility of EBS using EIOS to ensure timely detection of potential public health threats at the national level, (*12*) in alignment with information about country experiences presented by Brazil, Egypt, Iraq and Sierra Leone during the EIOS Global Technical Meeting in 2022. (*13*) In addition, five events posted on EIS from June to October 2023 were not identified as events following verification and daily assessment by the KDCA. This indicates that there may be variations in the standards used to identify events that reflect differences in the prioritization and potential impact of public health threats between the national and international levels.

Out of a total of 99 945 sources pulled into the EIOS dashboard from 1 June to 31 October 2023, 2844 were classified as signals and 425 as events. This implies a need for human intelligence to filter out irrelevant or duplicate articles from the substantial volume of data in EIOS. While human monitoring by experienced health professionals remains crucial when performing EBS activities using EIOS, such as building new dashboards to reflect monitoring for different priorities and reviewing detected events for verification, future research could explore the potential application of advanced artificial intelligence to improve the performance of EIOS by reducing noise – that is, irrelevant information about events of interest – and enhancing the accuracy of signal identification in EIOS.

In conclusion, despite the recognized need to reduce noise and expand global utilization of EIOS, using EIOS for EBS activities has proven to be efficient in aiding the early detection of potential public health threats at the national level, as reported from Republic of Korea. However, a systematic evaluation of EBS activities that use EIOS at the KDCA is required to identify relevant attributes, such as timeliness or sensitivity. (*14*)
